# Obituary

**Published:** 1888-08

**Authors:** Geo. L. Field


### Obituary.

At a called meeting of the Dentists of Lexington, Ky., to take action on the death of Dr. Stoddard Driggs, upon motion of Dr. T. D. Kelley, Dr. A. O. Rawls was called to the chair and the following resolutions were passed:

WHEREAS, Divine Providence has taken from our midst our friend and co-laborer, Dr. Stoddard Driggs:

*Resolved*, That we earnestly and most sincerely sympathize with the family of our deceased brother in this hour of their sad bereavement.

*Resolved*, That in the death of Dr. Driggs the community in which he lived so long, and favorably, and labored so patiently and faithfully, has lost one whose place will indeed be difficult to fill and that the Dental Profession at home and abroad has sustained the loss of one of its most skillful, earnest, and conscientious members; one of whom it can be well said he was true to noble convictions, true to the interests of his numerous patrons, and a just and exemplary *frater* to those of his calling.

*Resolved*, That we attend in body the funeral ceremonies of our departed friend and confrere and that a copy of these resolutions be given to the family of the deceased; also a copy to each of our city papers, and Dental Journals for publication.

T. D. KELLEY,
J. H. FLOORE,
S. S. JOHNSON,
F. B. BOSWORTH,
*(Committee.)*

---

DR. RICHARD W. ZIERLEIN, died in Washington, Missouri, on June 4th, 1888, of meningitis, at fifty years of age. He began the study of Dentistry with Dr. H. J. McKellops, in St. Louis, (the city of his nativity), in 1852. In 1865 he removed to Washington, Mo., where he opened an office for the practice of his profession, and where he remained until the time of his death. Naturally of a retiring disposition he took but little part in the meetings of his professional brethren; consequently was but little known outside of his own State. Gentle and kind-hearted as a woman, he above all things, loved his quiet country home. A loyal friend, a loving husband and father, his sudden taking away will leave a sadness in the hearts of many that will be deeply felt. He leaves a widow and two lovely daughters, two and four years of age, by whom he was followed to his last resting place in Bellefountain Cemetery, St. Louis.

GEO. L. FIELD, D.D.S.

